# *“Not just labelling medicines”*: Pharmacists' perspectives on their potential roles within youth mental health services

**DOI:** 10.1016/j.rcsop.2026.100723

**Published:** 2026-02-25

**Authors:** Phoebe M. Downey, Jack C. Collins, Sarira El-Den, Sara S. McMillan, Blake Hamilton, Donna Fowler, Connie M.S. Janiszewski, Sanam Fathabadi, Claire L. O'Reilly

**Affiliations:** a The University of Sydney School of Pharmacy, Faculty of Medicine and Health, The University of Sydney, Sydney, New South Wales, Australia; b School of Pharmacy and Medical Sciences, Griffith University, Gold Coast Campus, Southport, Queensland, Australia; c Centre for Mental Health, Griffith University, Nathan, Queensland, Australia; d Brain and Mind Centre, The University of Sydney, Camperdown, New South Wales, Australia

**Keywords:** Mental health services, Adolescent, Pharmacists, Qualitative research, Mental disorders

## Abstract

**Background:**

Youth mental illness is a significant global health concern, with increasing rates of psychological distress and unmet care needs. Despite their expertise in psychotropic medication management, pharmacists remain underutilised in youth mental health services, leaving their potential contributions to multidisciplinary care largely unexplored.

**Method:**

Semi-structured interviews were conducted with Australian pharmacists to explore their perspectives on medication use among young people and their potential roles in youth mental health services. Interviews were transcribed verbatim and analysed using reflexive thematic analysis.

**Results:**

Eighteen pharmacists from diverse practice areas shared their insights, generating four key themes: (i) *The Struggle for Equitable Access,* highlighting systemic barriers to health service access; (ii) *Medication as a Pillar, Not the Panacea,* advocating for balanced psychotropic medication use alongside psychosocial interventions; (iii) *Breaking the Dispensing Box,* revealing pharmacists' aspirations to expand their roles beyond dispensing through greater clinical involvement; and (iv) *Navigating Trust and Stigma,* discussing the challenges of building trust with young people amid stigma.

**Conclusions:**

Pharmacists are well-positioned to play a more integral role in youth mental healthcare by leveraging their expertise in medication management. However, role ambiguities, professional hierarchies, and stigma may restrict their contributions beyond “*just labelling medicines”*. Clear role definitions, collaborative frameworks, and targeted education are essential to empower pharmacists to engage in multidisciplinary care, while addressing systemic barriers is critical to integrating them into youth mental health services and enhancing holistic care.

## Highlights

• Pharmacists possess expertise in psychotropic medication management but are currently not integrated into youth mental health services in Australia.
• Participants expressed a desire for expanded roles, advocating for deeper clinical involvement and integration into multidisciplinary teams.
• Participants felt systemic barriers can restrict equitable access to youth mental healthcare and exacerbate challenges in service delivery.
• Role ambiguity and professional hierarchies were considered key barriers, emphasising the need for clear role definitions.

## 1 Introduction

Mental illnesses and substance use disorders are leading causes of disease burden and disability for young people worldwide, with 12% of those aged 5–24 years estimated to live with mental illness.^1^ For young people, the challenges of mental illness may be exacerbated by pervasive stigma, discrimination, and frequent human rights violations, which not only hinder their ability to seek help but also contribute to social isolation and worsening mental health.^2^ Over the past two decades, the mental health of young people has continued to decline, a concerning trend intensified by the COVID-19 pandemic.^3^, ^4^, ^5^

In response to the demand for improved youth mental healthcare, many countries are establishing and transforming youth mental health services into holistic and accessible places of care.^6^ This shift acknowledges the growing understanding that multidisciplinary early intervention is essential for effective management of youth mental health.^7^, ^8^ One such initiative is *headspace*, an early intervention service established by the Australian government in 2006, which seeks to improve access and service cohesion for young people experiencing mental and substance use disorders.^9^ In Australia, while many primary care and mental health services are partially or fully subsidised through Medicare, patients may still encounter variable out-of-pocket costs depending on billing practices. In contrast, *headspace* services are typically delivered at low or no cost for Medicare card holders, providing a more accessible option for young people experiencing mental distress.^10^

Amid this broader response to the youth mental health crisis, there has been a marked increase in the use of psychotropic medications. In Australia, the rate of psychotropic prescribing among individuals under 18 years of age doubled between 2013 and 2021.^11^ Despite the increasing use of these medications, the evidence base for their use in young people remains limited, particularly regarding long-term outcomes.^12^, ^13^, ^14^, ^15^ This limited evidence, along with concerns about overprescribing,^16^ emphasises the need for further research and the adoption of innovative, integrated treatment strategies. As medication experts, pharmacists are uniquely positioned to advocate for the safe and effective use of psychotropic medications, potentially playing a key role in alleviating the burden of mental illness among young people.^17^

Despite the potential roles pharmacists could play in mental healthcare, their reported involvement in youth mental health settings is currently limited. Prior Australian studies suggest that pharmacists could be crucial in supporting young people with medication management,^18^, ^19^ while UK evaluations have shown that embedding pharmacists within child and adolescent mental health services can enhance medication management and deliver cost savings.^20^ Recent Australian research has also demonstrated that staff within youth mental health services recognise the valuable contribution pharmacists could make in optimising medication use, although barriers such as role ambiguity and limited awareness of pharmacists' full scope may hinder effective integration.^21^ However, existing research has largely focused on young people's medication experiences and staff expectations of pharmacist involvement, providing limited understanding of how pharmacists themselves conceptualise their potential role within youth mental health services. Given that services such as *headspace* present distinct clinical and relational demands that differ from traditional pharmacy practice, understanding pharmacists' own perspectives is essential to inform feasible models of integration. Therefore, this study aimed to explore pharmacists' perspectives on medication use among young people accessing youth mental health services and their potential roles within these services.

## 2 Methods

Ethical approval was obtained from The University of Sydney Human Research Ethics Committee (HREC Approval No. 2023/454). This study adheres to the Consolidated Criteria for Reporting Qualitative Research (COREQ) checklist.^22^

### 2.1 Study design and recruitment

This exploratory study employed semi-structured interviews to explore pharmacists' perspectives on their current and potential roles in youth mental healthcare and medication management. Participants were recruited via convenience sampling through direct outreach to professional pharmacy organisations, social media, and research team member networks from August to September 2024. Interested registered Australian pharmacists completed an ‘Expression of Interest’ survey via REDCap, an electronic data capture tool hosted by The University of Sydney.^23^, ^24^ Respondents were contacted via email to confirm participation and schedule interviews; non-responses after three follow-up attempts were not pursued further. Participants provided written and verbal consent prior to participation and received an AUD 50 gift voucher upon completing the interview.

### 2.2 Data collection

An interview guide (Supplementary Material) was developed based on relevant literature^18^, ^19^, ^20^ and refined through pilot testing with three volunteers with a background in pharmacy. Minor adjustments were made to enhance interview flow and clarity. These pilot interviews were excluded from the final analysis. Interviews were conducted online via Zoom® (Zoom Video Communications, Inc., San Jose, USA) by PD between August to September 2024. Interviews were audio-recorded and transcribed verbatim using Zoom's ‘Live Transcription’ feature, followed by a thorough review for accuracy, including manual corrections by PD where required.

### 2.3 Data analysis

Reflexive Thematic Analysis (RTA) was chosen for its flexibility in capturing both the subjective experiences of pharmacists and the broader systemic challenges they navigate.^25^ RTA was particularly appropriate for this study, acknowledging the researcher's active role in constructing themes through data engagement, rather than passive identification—a reflexivity essential given PD's positionality as both a female pharmacy student with community pharmacy experience and as a young person with experience navigating the healthcare system. This dual perspective may have influenced both data generation and analysis, as participants may have assumed a shared understanding, although no relationship was established with participants prior to study commencement. For example, PD's familiarity enabled deeper questioning of barriers to youth-specific services, and personal experiences shaped interpretation of themes related to accessibility and trust. A constructivist orientation underpinned this research, recognising that the insights of pharmacists interviewed were co-constructed through their interactions with the healthcare system and the young people they serve.^26^ The analysis was primarily inductive, with themes grounded in participants' experiences and generated through careful engagement with the data.^25^, ^27^

The analysis followed Braun and Clarke's six-phase approach with iterative engagement throughout the process.^28^ Familiarisation began during transcription and was deepened through repeated readings of the transcripts. NVivo 14 (Lumivero LLC, Denver, USA) was used to code the transcripts, applying semantic codes to capture explicit meanings and latent codes to identify underlying patterns in the data. An initial thematic map was developed to visually represent interrelationships among these codes and was iteratively revisited to capture emerging insights. While many links were directly grounded in participants' accounts, others reflect interpretive connections informed by reflexive engagement with the data. Theme refinement was a collaborative process, with a second researcher (COR)—a female pharmacist, experienced mental health educator and mental health academic—familiarising herself with 25% of the transcripts, and regular reflexive discussions within the team to ensure theme depth and coherence.

## 3 Results

**Fig. 1:**
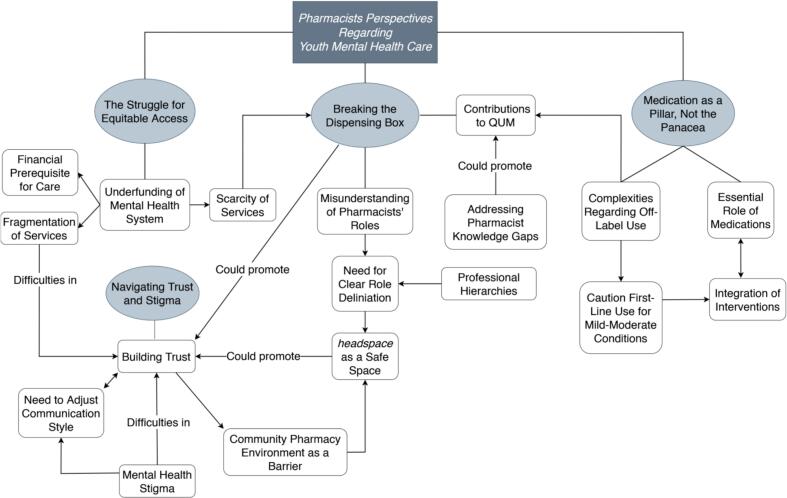
Thematic map showing relationship between themes and codes generated from interviews. Note: Unidirectional arrows indicate a one-way influence, whereas bidirectional arrows indicate mutual interaction.

Of the 34 eligible expressions of interest received, 18 pharmacists participated in interviews (mean duration: 34 min, range: 20 to 54 min) (Table 1). Four overarching themes were generated from the data: (i*) The Struggle for Equitable Access*, (ii) *Medication as a Pillar, Not the Panacea*, (iii) *Breaking the Dispensing Box*, and (iv) *Navigating Trust and Stigma* (Fig. 1).

**Table 1:** Characteristics of pharmacists interviewed (*n* = 18).

Characteristics	Frequency (n)	Percent (%)
Gender		
Female	17	94.4
Male	1	5.6
Age		
20–29	5	27.8
30–39	10	55.5
40–49	3	16.7
State/territory		
Australian Capital Territory	2	11.1
New South Wales	11	61.1
Queensland	1	5.5
South Australia	1	5.5
Victoria	2	11.1
Western Australia	1	5.5
Self-described primary location of practice		
Metropolitan	14	77.8
Regional	3	16.7
Rural	1	5.5
Years experience		
< 5	5	27.8
5–10	7	38.9
11–15	3	16.7
16–20	2	11.1
>20	1	5.5
Self-described main area of practice		
Community	6	33.3
Hospital	8	44.4
Non-patient facing roles⁎	4	22.2

⁎ Examples of non-patient facing roles include roles in digital health, the government sector, and trial implementation.

### 3.1 The struggle for equitable access

Participants described a range of systemic barriers restricting young people's access to mental healthcare, particularly those related to limited service availability and broader infrastructure shortcomings. These obstacles, they observed, reflect the complex realities of balancing a commitment to equitable care with system constraints.

Foremost among these obstacles was the scarcity of mental health services, particularly in rural and regional settings. Those with firsthand experience working in these areas recounted how patients often lived “*really far away, and even the closest health clinic… is maybe at least an hour away*” (P05). Such isolation, participants warned, can leave young people without necessary support and at risk of “*fall*[ing] *through the cracks”* (P13). Nation-wide provider shortages were thought to further exacerbate these disparities, forcing young people into lengthy wait periods and raising the risk that their conditions will deteriorate before any treatment commences. With specialists “*often* [having] *limited hours… and limited space within those hours”* (P07), accessing necessary care can become a prolonged ordeal. These delays were said to exacerbate mental health conditions, as *“things can get worse”* (P16) without timely support.

Participants also pointed to how financial barriers can obstruct access to care, particularly for those from lower socioeconomic backgrounds. Many remarked that the “*incredibly high cost*” (P13) of mental health specialists can render care inaccessible, with many young people “[unable to] *afford to see* [a] *psychiatrist”* (P07), leading to delays or a complete inability to access care. Although pharmacists, often more accessible than other healthcare professionals, described stepping in to bridge the gap when specialist care was unattainable by addressing *“logistical issues… to make sure that all mental health patients have access to medication in a timely manner”* (P05), they reported that some young people simply “*r*[an] *out of money”* (P05) and were consequently forced to forgo their medication. This financial prerequisite for care, they warned, leaves vulnerable young people with limited or no support at all, undermining their chances of receiving adequate, ongoing care.

Concerns about fragmented services emerged as yet another systemic shortcoming that magnified the effects of scarcity and high costs. Participants frequently described the lack of coordination between mental health providers, pushing young people to navigate disconnected pathways with little guidance. The “*lack of follow-up*” (P07) and poor communication, particularly between hospital and community care, led young people to “*com*[e] *in and out, in and out*” (P05) of services, sometimes recounting traumatic experiences repeatedly and “*caus*[ing] *additional trauma*” (P01). Within this disjointed landscape, some participants noted that many families lacked awareness of “*what organisations are available, where they are, how to get there*” (P09) or *“where* [they] *should… go to get support*” (P13). This interplay of structural fragmentation and limited fallback options amplified the challenge of accessing care, at times delaying intervention until problems became *“really, really bad”* (P13) and leaving pharmacists feeling powerless to provide truly cohesive care.

Underlying many of these hurdles was the perceived chronic underfunding of mental health services, limiting the system's capacity to respond to growing demand and intensifying the strain on existing structures. The struggle for sufficient resources, encapsulated by the sentiment “*it's always going to be money”* (P15), reflected frustrations among participants who felt limited in their ability to provide optimal care without adequate support. Underfunding, coupled with limited career advancement and poor remuneration, left pharmacists *“disheartened”* (P15) and contributed to workforce attrition, described as a *“waste of potential and talent”* (P05), further shrinking the pool of accessible mental health providers. This chronic underfunding was said to not only compromise service quality but also erode the morale and sustainability of mental health provision.

### 3.2 Medication as a Pillar, Not the Panacea

Pharmacists interviewed reflected on the role of medication in youth mental health, recognising its importance while simultaneously grappling with concerns surrounding off-label use and potential side effects. Participants acknowledged the complexities of prescribing psychotropic medications to young people, particularly considering the limited evidence base for many of these treatments. They felt this paucity of robust data could compel clinicians to proceed despite *“not [much] strong evidence*” (P16). Off-label use was seen as a particularly delicate issue, with adolescence described as a uniquely vulnerable period “*hormonal changes and imbalances* [that] *can also affect… their mental health states”* (P07). Decisions were often framed as requiring nuanced, individualised care, grounded in “*what's appropriate for that patient at that time*” (P17). Some cautioned against becoming “*moralistic and alarmist”* (P17) and approaching medication use through an “*all-or-nothing”* lens (P17), instead advocating for care that considers both the benefits and potential risks in the context of the young person's evolving needs and preferences.

Building on the importance of an individualised approach, participants consistently acknowledged the essential role that medications can play in youth mental healthcare, particularly in ensuring young people reach a stable state where they can *“follow up with other non-medication treatments”* (P03). Participants were generally supportive of using medications in conditions that they associated with a *“chemical imbalance where* [medication] *can help with that chemical imbalance”* (P15). For instance, psychostimulants for attention-deficit/hyperactivity disorder (ADHD) were praised as their *“evidence base is massive,* [the] *effect size is huge, and safety is excellent”* (P17). Similarly, antipsychotics were recognised for their ability to manage symptoms of psychosis and *“de-escalate the scenario”* (P08). However, for mid-to-moderate anxiety or depression, many saw medication as *“a last resort”* (P11), citing potential adverse effects such as feeling *“less cognisant and less… like themselves”* (P07). Despite these reservations, participants acknowledged that timely pharmacological intervention could be *“so important… because* [it] *can prevent the negative outcomes that come from not treating”* (P10), helping to stabilise mental health and allow engagement with further supports.

While accepting the value of pharmacotherapy, participants strongly advocated for non-pharmacological interventions as the preferred first-line treatments for young people, particularly in mild to moderate conditions. Counselling, behavioural therapies, and lifestyle interventions were consistently emphasised, alongside broader community support. This focus on creating a sense of *“connection, an outlet …, and a sense of belonging*” (P12) was seen as integral to sustaining mental health and reducing unnecessary medication use. Participants emphasised that treatment should involve *“therapy first, rather than putting them on medications straight away*” (P11), with medication introduced only as a complementary tool in a more expansive toolkit aimed at promoting long-term wellbeing.

### 3.3 Breaking the dispensing box

Frustrated with traditional dispensing constraints, participants voiced a desire for deeper involvement in youth mental healthcare. They saw the potential to expand their roles beyond the boundaries of medication supply by participating in clinical decision-making, applying their expertise collaboratively within multidisciplinary teams, ultimately striving for more integrated support for young people.

Emphasising their capacity to *“support the safer, quality use of medicines”* (P02), many participants highlighted their expertise in assessing treatment appropriateness and managing complex regimens. Their deep pharmacological knowledge was considered vital in understanding *“what the body does to the medication and what the medication does to the body and the mind”* (P07), enabling them to guide both young people and healthcare professionals through intricate treatment plans. Some also saw opportunities to contribute to deprescribing pathways or early interventions that might *“negate the need for medicines*” (P04), reflecting a desire to participate proactively within a multidisciplinary context.

Despite these aspirations, perceived professional hierarchies were feared to constrain pharmacists' contributions, limiting their involvement in broader therapeutic discussions. Many participants expressed frustration over the reduction of their role to *“just put*[ting] *a label on a box”* (P15), a simplification that neglected their clinical expertise. Some attributed this to a broader lack of understanding about pharmacists' skill sets, observing that many healthcare professionals *“don't know how to use pharmacists”* effectively (P01). To address these challenges, integrating pharmacists within multidisciplinary services was suggested as a way to “*help others appreciate our role”* (P07) given that “*once* [healthcare services] *get a pharmacist, they don't want to lose the pharmacist*” (P02).

Clear role definitions were deemed essential to promoting efficient collaboration and avoiding conflicts. They emphasised that a cohesive team environment where “*each individual… understands the nature and purpose of each role*” (P01) would be crucial in optimising care and ensuring harmonious collaboration. Some cited the need for *“very clear professional autonomy over the pharmacotherapeutic side of things”* (P07), believing that specifying responsibilities from the outset would avert *“stepping on the toes of existing roles”* (P11), ease early-stage *“teething issues*” (P04), and create an environment that values each professional's unique skills, thereby promoting a collaborative approach to patient care.

Some participants also acknowledged the need to address their own knowledge gaps to contribute effectively to youth mental healthcare. Familiarity with youth mental health services and referral pathways varied considerably; while some, particularly those with mental health experience, demonstrated a nuanced understanding of these networks, others admitted to having limited knowledge, remarking that it was *“a little bit embarrassing”* (P03). This inconsistency emphasises the importance of “*finding the right fit”* (P01) when integrating pharmacists into services like *headspace*. To truly fulfil their potential in these roles, some participants called for targeted education beyond “*just Mental Health First Aid*”^e^ (P10), focusing on the skills needed for effective support. Mental Health First Aid® (MHFA®) training, an evidence-based training program to support skills, confidence and preparedness to provide mental health support is increasingly being embedded in pharmacy curricula.^29^ However, it was viewed by several participants as insufficient preparation for the complexities of youth mental health practice. Embedding trained pharmacists in these settings was seen as an opportunity to build trust and enhance their role within multidisciplinary teams, ultimately improving support for young people.

### 3.4 Navigating trust and stigma

The pharmacist-patient relationship was a central focus in participants' discussions, with trust identified as a cornerstone of effective mental health support. Participants noted that stigma surrounding mental illness, combined with environmental constraints in community pharmacy settings, often created barriers to fostering trust with young people.

Building meaningful connections with young people was seen as particularly challenging in community pharmacy settings, where limited privacy and time constraints often made it difficult to nurture the trust and sense of safety necessary for open discussions about mental health. Participants reflected on how these “*fast-paced, busy environment*[s] [acted as] *barrier*[s] *to relationship building”* (P03), preventing young people from feeling comfortable enough to share their concerns, especially when “[unsure of] *how the pharmacist feels about mental health”* (P09). This tension between pharmacists' desire to offer support and the practical limitations of their role recurred throughout the interviews. In contrast, services like *headspace* were perceived as more conducive to trust-building; their one-on-one consultations in a “*non-judgemental place”* (P07) enabling deeper conversations than typically possible in community pharmacies.

Extending beyond physical and logistical constraints, participants also identified mental health stigma as a powerful deterrent, undermining young people's willingness to seek help. Participants observed that young people often seemed *“hyper-aware”* (P09) of how others perceived them, especially in public spaces like community pharmacies. This heightened awareness, combined with pre-existing stigma about mental illness and medication, was described as major deterrent to accessing care from pharmacists. It was noted that many young people needed reassurance that “*it's okay to be on medication*” (P05), indicating the pervasive nature of medication stigma and its impact on young people's willingness to engage with treatment. In youth mental health services, participants saw opportunities to *“reduce the fear or the stigma in asking healthcare professionals for help”* (P01), positioning pharmacists as accessible and trusted allies in young people's healthcare journeys.

Still, maintaining both professional authority and accessibility remained a delicate balancing act. Some participants felt their status as *“not the doctor”* (P14) opened avenues for candid conversations, although they acknowledged the need to adapt communication styles—being “*understanding, compassionate, respectful, mindful, thoughtful, sensitive”* (P01)—to ensure young people were *“include*[d] *in that discussion”* (P12). Integration into youth mental health services like *headspace* was viewed as an opportunity to forge deeper connections, positioning pharmacists as accessible medication experts within a supportive environment. By fostering inclusive communication, it was posited that pharmacists could promote “*a lot more knowledge and a lot more appreciation of… how pharmacists can help”* (P7), potentially reshaping how young people interact with pharmacists throughout their healthcare journey.

## 4 Discussion

This study explored the perspectives of pharmacists concerning their roles in youth mental healthcare, highlighting both their commitment to improving outcomes for young people and the substantial systemic and professional barriers they encounter. As an area largely unexplored in previous research, these accounts demonstrate a strong dedication to accessible and equitable care, yet pharmacists' enthusiasm was tempered by challenges such as fragmented service delivery and resource limitations. In alignment with prior research on mental healthcare access,^30^, ^31^ they emphasised the decisive influence of social determinants, such as rurality and socioeconomic status, on young people's ability to obtain timely, appropriate support.^32^ These underlying constraints shaped subsequent reflections on medication, professional integration, and the delicate process of building trust with young people.

Central to their concerns was the persistent underfunding and limited integration of pharmacists within the broader healthcare system. Despite pharmacists being among the most accessible healthcare professionals,^33^ many participants witnessed how underfunding and limited integration within the healthcare system impede their ability to offer comprehensive support.^34^, ^35^ This feeling of underutilisation can not only affect participants' professional satisfaction but also perpetuate existing health inequities by limiting access to timely interventions and medication management, areas where participants believed they could make a substantial impact. Notably, pharmacists in this study emphasised these systemic barriers unprompted, indicating the deep entrenchment of these challenges in their professional experience. Their collective frustration mirrors broader concerns about workforce dissatisfaction and burnout among healthcare professionals,^36^, ^37^ where feelings of powerlessness can exacerbate burnout and contribute to provider shortages—a vicious cycle that further strains the healthcare system.^38^, ^39^, ^40^ These dynamics suggest that structural and workforce conditions more strongly shape pharmacists perceived capacity to contribute within youth mental healthcare, rather than reflecting a lack of willingness or interest among pharmacists themselves.

Encouragingly, evidence suggests that when pharmacists are integrated into multidisciplinary teams, they can improve health outcomes. For instance, in the UK, pharmacists embedded in child and adolescent mental health services have enhanced medication safety and delivered cost savings.^20^, ^41^ While regulatory frameworks vary, with the UK granting pharmacists greater scope in independent prescribing,^42^ similar integration in multidisciplinary settings in Australia has yielded promising results. For example, in an Australian Mental Health Hospital-in-the-Home setting, integrating a clinical pharmacist improved medication safety through effective reconciliation and adverse drug reaction management.^43^ Likewise, pharmacist integration into a shared-care model in a paediatric disability service reduced appointment demands, shortened wait times, improved the timeliness of medication reviews, and achieved a return on investment.^44^ These examples highlight the potential for pharmacists to bridge healthcare gaps for young people, particularly in improving medication safety, reducing costs, and supporting better access to psychotropic medicines.

Nevertheless, participants cautioned against an over-reliance on psychotropic medication, while acknowledging its critical role improving symptoms and quality of life in young people, as seen with psychostimulants in managing ADHD,^45^, ^46^ and antipsychotics like risperidone and aripiprazole for the treatment of schizophrenia and behavioural conditions.^13^ However, robust evidence on long-term outcomes in young people remains limited.^14^, ^47^, ^48^, ^49^ Participants acknowledged the limitations of relying solely on pharmacological interventions, stressing that medications, while at times valuable, are often insufficient to address the broader social and psychological dimensions of mental health.

Building trust with young people was identified as another key challenge, particularly in community pharmacy settings where interactions are often brief and transactional. Trust is a fundamental component of effective healthcare,^50^, ^51^ and its absence can hinder patient engagement and adherence to treatment.^52^ Pharmacists interviewed noted that stigma surrounding mental illness, combined with the fast-paced nature of community pharmacies, made it difficult to create non-judgmental spaces for open dialogue, a challenge corroborated by existing research.^53^, ^54^ The fragmentation of services, where young people are passed between different providers without continuity of care, further impedes the development of trusting relationships. This discontinuity, characterised by a lack of communication between healthcare professionals and insufficient service integration, can leave patients feeling unsupported and disengaged as they struggle to navigate the system without adequate guidance.^55^ Pharmacists in this study contrasted community pharmacy with *headspace*, describing the latter as a setting where they could provide in-depth consultations, help destigmatise medication use, and position themselves as accessible allies in patients' mental health journeys.

While *headspace* has been shown to deliver valuable early intervention services, with over 50% of *headspace* clients reported to demonstrate clinically significant improvements in either psychological distress, functioning, or quality of life,^56^ its' current service model is not designed to address the needs of young people with higher levels of need. For instance, Cross et al. found that clients with more complex needs, experienced only modest improvements despite additional services and frequent prescription of psychotropic medications.^57^ This shortfall is especially concerning given estimates that 50–60% of young people presenting at headspace have moderate to high clinical complexity.^58^ Within this context, embedding a pharmacist, as envisaged by participants, may enhance service responsiveness by strengthening medication safety, adherence, and shared decision-making through counselling, psychoeducation, and medication review.^17^ Operationalising such a role would require paediatric psychopharmacology knowledge and communication skills informed by trauma-informed and culturally safe practice.^59^ Comparable models overseas have demonstrated safety and economic benefits,^20^ yet pharmacist involvement in youth mental healthcare is not yet routine in Australian healthcare settings. Future research should focus on piloting and evaluating non-dispensing pharmacist roles within multidisciplinary youth mental healthcare settings to generate evidence for the potential contribution of pharmacists to improving the quality use of medicines for young people living with mental illness.

### 4.1 Strengths and limitations

A key strength of this study lies in its focus on pharmacists' own perspectives, a lens that has been largely absent from existing work on youth mental healthcare. The use of RTA supported close attention to how pharmacists understood their roles to be shaped by contextual and relational conditions within youth mental health services. However, several limitations must be acknowledged. The sample was predominantly female and based in metropolitan New South Wales, which may have shaped how pharmacists understood and described their potential role within youth mental healthcare. This demographic concentration, together with the use of convenience sampling that may have favoured pharmacists with prior interest or experience in mental health, limits the transferability of the findings to broader pharmacy settings. Furthermore, the focus on the Australian healthcare system limits the generalisability of its findings to other contexts where pharmacist scope and regulatory frameworks differ. Future research should incorporate perspectives from other stakeholders, including young people, and draw on diverse pharmacist samples to provide a more comprehensive understanding of pharmacists' integration into youth mental health services.

## 5 Conclusion

This study offers insights into how pharmacists view their potential roles in youth mental healthcare, particularly in relation to psychotropic medication management, education, and multidisciplinary collaboration. Participants described both enthusiasm for deeper involvement and the structural, professional, and relational barriers that may constrain these contributions. Role ambiguity, professional hierarchies, and stigma shaped pharmacists perceived capacity to engage with young people and meaningfully support their case. These findings suggest that clearly defining pharmacists' roles and developing stronger collaborative frameworks may support cohesive medication-related care. Future research should actively involve young people and caregivers to explore how pharmacists can be better integrated into youth mental health services in ways that address their needs.

## Study funding

This research was supported by the Bright Ideas Grant from The University of Sydney Faculty of Medicine and Health. PD was supported by the Andrew Tu Scholarship in Pharmacy.

## CRediT authorship contribution statement

**Phoebe M. Downey:** Writing – review & editing, Writing – original draft, Visualization, Investigation, Formal analysis, Data curation. **Jack C. Collins:** Writing – review & editing, Supervision, Methodology, Conceptualization. **Sarira El-Den:** Writing – review & editing, Supervision, Funding acquisition, Conceptualization. **Sara S. McMillan:** Writing – review & editing, Methodology. **Blake Hamilton:** Writing – review & editing, Project administration. **Donna Fowler:** Writing – review & editing, Project administration. **Connie M.S. Janiszewski:** Writing – review & editing, Project administration. **Sanam Fathabadi:** Writing – review & editing, Methodology. **Claire L. O'Reilly:** Writing – review & editing, Supervision, Project administration, Methodology, Funding acquisition, Formal analysis, Conceptualization.

## Declaration of competing interest

Three co-authors work at *headspace* but in line with our Ethics approval were not directly involved in the recruitment, data collection, or analysis processes of this study.

## Appendix A Supplementary data

Supplementary material Key components of the interview guide Image 1

## Footnotes

e Mental Health First Aid® (MHFA) is an accredited training course designed to equip people with the knowledge, skills and confidence to recognise, understand and respond to someone experiencing a mental health problem or mental health crisis (https://www.mhfa.com.au/)
