# Supplementary material for: “Not just labelling medicines”: Pharmacists' perspectives on their potential roles within youth mental health services
Source: Explor Res Clin Soc Pharm. 2026 Feb 25;22:100723. doi: 10.1016/j.rcsop.2026.100723 (PMC12972700; doi:10.1016/j.rcsop.2026.100723)
Supplement: Supplementary file 1 — Supplementary material Key components of the interview guide [file mmc1.docx]

**Supplementary material:** Key components of the interview guide.

| **Main topic** | **Key questions/prompts** |
| --- | --- |
| Personal and professional demographics | - Years of experience - Main area of practice |
| Pharmacists’ perspectives on their role in managing and supporting the mental health of young people | - Role in treatment and management - Sources of support and referral - Awareness and use of youth mental health services as a referral pathway - Role of medications - Decision making and information |
| Pharmacists' potential roles in youth mental health services | - Current roles in youth mental healthcare - Barriers and facilitators of potential roles in youth mental health services - Impact and value of potential roles in youth mental health services |
